# Patterns of Recurrence and Survival Rate After Complete Resection of Pathological Stage N2 Small-Cell Lung Cancer

**DOI:** 10.3389/fonc.2021.675354

**Published:** 2021-08-27

**Authors:** Lian Yu, Jianlin Xu, Rong Qiao, Hua Zhong, Baohui Han, Runbo Zhong

**Affiliations:** Department of Pulmonary Medicine, Shanghai Chest Hospital, Shanghai Jiaotong University, Shanghai, China

**Keywords:** small cell lung cancer, limited disease, surgery, mediastinal lymph nodes, prognosis

## Abstract

The benefits of surgical resection for patients with stage N2 limited-disease small-cell lung cancer (LD-SCLC) remain controversial. This retrospective study analyzed the survival and recurrence patterns of the patients diagnosed with pathological N2 (p-N2) LD-SCLC after radical resection. A total of 171 p-N2 LD-SCLC patients who underwent radical pulmonary resection and systematic lymphadenectomies at Shanghai Chest Hospital from July 2005 to June 2015 were enrolled. The influence of the mediastinal lymph node status (single or multiple nodes, single- or multiple-station) on the survival and recurrence patterns was retrospectively analyzed. The main recurrence sites were outside the chest cavity (54.8%) and hematogenous metastasis (67.4%). The bone and liver as initial recurrence sites had a poor prognosis, with a median overall survival (OS) of 13.100 months and 11.900 months, respectively. The median disease-free survival (DFS) of patients diagnosed with single and multiple p-N2 after surgery were 19.233 and 9.367 months (P = 0.001), and the median OS were 43.033 and 17.100 months (P < 0.001), respectively. In conclusion, recurrence occurred in the form of hematogenous metastasis mostly in the extra-thoracic part. Interestingly, patients diagnosed with single p-N2 benefited from radical resection. Surgery may be a treatment option regardless of the T stage if N2 SCLC with a single metastatic lymph node can be identified preoperatively.

## Introduction

Although tobacco-control measures have led to a decline in the incidence and mortality of lung cancer over the past decades, this cancer remains to be among the leading causes of cancer mortality worldwide (1, 2). Small-cell lung cancer (SCLC) accounts for 15% of all lung cancers, with a low (7%) 5-year survival (3). The disease is characterized by rapid tumor growth and early metastasis, associated with poor prognosis (4).

Two randomized phase III studies conducted in the 1960s (5) and the 1980s (6) reported negative results with surgery in limited-disease SCLC (LD-SCLC) patients and, thereafter, surgery has been discouraged. The aforementioned studies were critical in shaping treatment recommendations for LD-SCLC. Currently, the International Guidelines highlight that surgery is justified for selected stage I (T1-2,N0M0) SCLC patients, after adequate staging. Concurrent chemoradiotherapy is the standard of care in the rest of limited disease SCLC (LD-SCLC) instead of radical surgery, especially in patients diagnosed with lymph node metastasis (N1-N2) (7). However, more investigations should be done to identify the applicability of surgery to modern practice. There are few studies focused on the lymph node status of the patients diagnosed with pathological N2 (p-N2) SCLC, whether they could benefit from radical surgery or chemoradiotherapy.

Several studies (8–12) have retrospectively analyzed the prognosis and survival of patients with LD-SCLC after surgery, involving a small number of patients with p-N2. We collected a large number of cases of p-N2 LD-SCLC patients diagnosed through surgery. This study aimed to analyze the patterns of recurrence and survival of 171 Chinese p-N2 LD-SCLC patients after complete resection. It focused mainly on the relationship between lymph node status and the overall survival of the patients.

## Material and Methods

### Patients

The medical history and clinical data of all patients diagnosed with SCLC through surgical pathology at Shanghai Chest Hospital, from July 2005 to June 2015, were obtained; a total of 350 cases were analyzed. There were 171 patients diagnosed with p-N2 after radical resection. EBUS-TBNA and PET-CT scan were not routinely performed as part of the preoperative staging work-up. Chest CT, abdominal B-scan ultrasonography, cranial CT or MRI, and bone scintigraphy were routinely performed, if the patients had not undergone PET-CT scan. The inclusion criteria were as follows: (i) surgically complete resection with systematic lymph node dissection adopted as a standard procedure; (ii) postoperative histopathology confirmed as p-N2 SCLC; (iii) clinical data and follow-up information were complete. The exclusion criteria were as follows: (i) patients who had incomplete resection, microscopically positive resections, and wedge resections; (ii) histological type other than SCLC. The Ethics Committee of Shanghai Chest Hospital reviewed and approved this study.

### Observed Indicators and Study Endpoints

According to the result of CT, B-scan ultrasonography every 3 months in the follow-up process, the site and time of recurrence at the first progression were recorded and analyzed. The disease-free survival (DFS) was calculated as the date of surgery to the disease progression or the last follow-up visit. The overall survival (OS) was determined as the date of surgery to death or last follow-up visit. The follow-up visit ended till death or November 20, 2020.

### Statistical Analysis

Data was statistically analyzed using SPSS26.0 statistical software (IBM, Armonk, NY, USA). Descriptive statistics were recorded in specific numbers, percentages, medians, and ranges. The DFS and OS were obtained using the Kaplan-Meier survival methods, whose statistical results were presented in the form of median DFS, median OS, and their respective 95% confidence intervals (CIs). The log-rank test was used to compare the DFS and OS of different characteristic groups. Multivariable Cox regression model was used to identify the significant factors related to DFS and OS. P < 0.05 was considered statistically significant.

## Results

### Patients, Clinical Characteristics, and Treatment

This retrospective study included 171 patients with p-N2 LD-SCLC who had undergone surgery. Among these patients, 140 (81.9%) were males and 113 (66.1%) were smokers. The median age was 59 years old, with a range from 33 to 76. All patients were clinically staged based on preoperative clinical data, including 12 c-IA patients (7.0%), 2 c-IB patients (1.2%), 1 c-IIA patient (0.6%), 38 c-IIB patients (22.2%), and 118 c-IIIA patients (69.0%). According to surgical pathology, there were 50 p-T1 patients, 77 p-T2 patients, 27 p-T3 patients, and 17 p-T4 patients, accounting for 29.2%, 45.0%, 15.8%, and 9.9%, respectively. Regarding lymph node status, the number and stations of the metastatic N2 lymph nodes were reviewed. Among these 171 patients, single p-N2 (n = 69) accounted for 40.4% of all and multiple p-N2 (n = 102) accounted for 59.6%. Single-station p-N2 (n = 102) accounted for 59.6% of all and multiple-station p-N2 (n = 69) accounted for 40.4%. At the last follow-up, 36 (21.1%) patients had not relapsed.

After the operation, except for 17.5% of patients who did not receive chest radiotherapy due to personal reasons, 141 patients (82.5%) received thoracic radiation therapy (TRT). Meanwhile, 54 (31.6%) received prophylactic cranial irradiation (PCI). Also, 149 patients (87.1%) received postoperative chemotherapy on record, with platinum-etoposide as the main regimen (**Table 1**).

**Table 1:** Characteristics of 171 patients.

Characteristic	No. of patients (%)
Age (years)	
Median	59
Range	33–76
<60	91 (53.2)
≥60	80 (46.8)
Gender	
Male	140 (81.9)
Female	31 (18.1)
Smoking status	
Never-smoker	58 (33.9)
Smoker	113 (66.1)
Preoperative (clinical) T stage	
1	52 (30.4)
2	102 (59.6)
3	13 (7.6)
4	4 (2.3)
Preoperative (clinical) N stage	
0	15 (8.8)
1	54 (31.6)
2	102 (59.6)
Preoperative (clinical) stage	
IA	12 (7.0)
IB	2 (1.2)
IIA	1 (0.6)
IIB	38 (22.2)
IIIA	118 (69.0)
Pathologic T stage	
1	50 (29.2)
2	77 (45.0)
3	27 (15.8)
4	17 (9.9)
No. of metastatic N2 lymph nodes	
Single	69 (40.4)
Multiple	102 (59.6)
Stations of metastatic N2 lymph nodes	
Single-station	102 (59.6)
Multiple-station	69 (40.4)
Adjuvant chemotherapy	
Etoposide and cisplatin	45 (26.3)
Etoposide and carboplatin	84 (49.1)
Other regimens	20 (11.7)
No chemotherapy	22 (12.9)
Adjuvant thoracic radiation therapy	
Yes	141 (82.5)
No	30 (17.5)
Prophylactic cranial irradiation	
Yes	54 (31.6)
No	117 (68.4)

### Patterns of Recurrence and DFS

**Figure 1:**
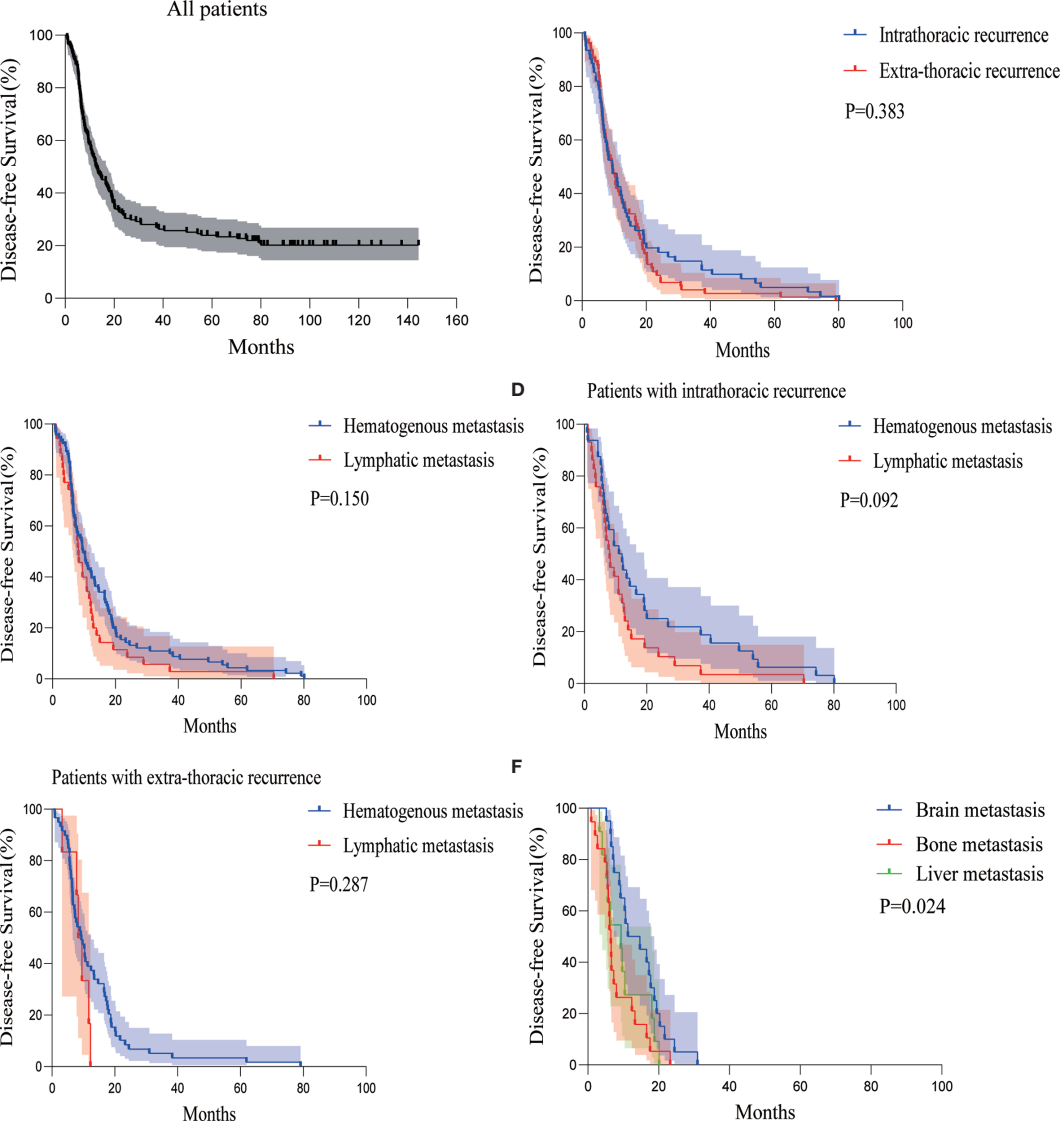
Kaplan-Meier curves of DFS for **(A)** all patients, **(B)** patients with intra- or extra-thoracic recurrence, **(C)** patients with hematogenous or lymphatic metastasis, **(D)** patients with intrathoracic recurrence in the form of hematogenous or lymphatic metastasis, **(E)** patients with extra-thoracic recurrence in the form of hematogenous or lymphatic metastasis, and **(F)** patients with brain, bone, or liver metastasis. DFS, disease-free survival.

**Figure 2:**
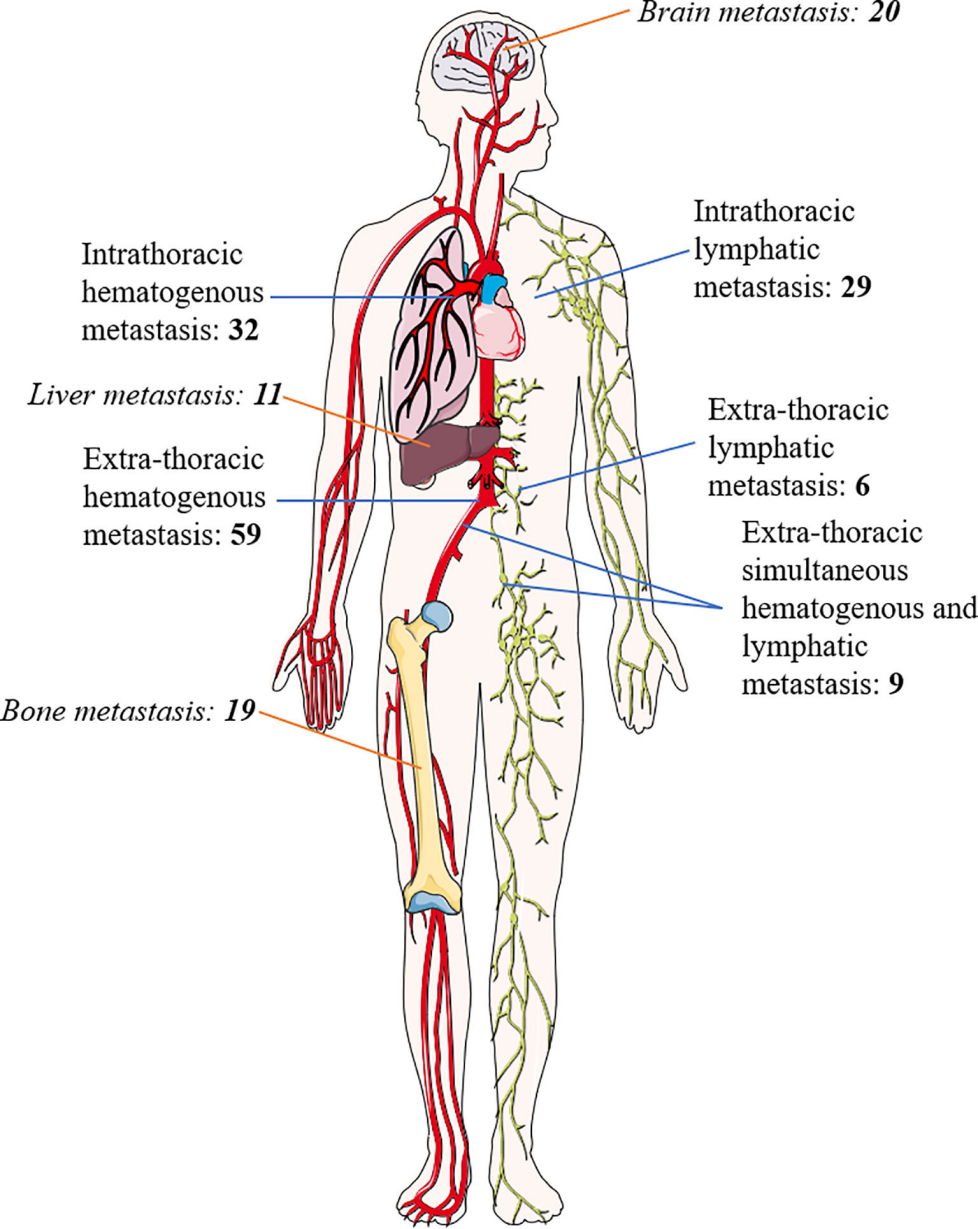
Recurrence patterns of all relapsed patients.

The median DFS of all the patients was 12.667 months (95%CI 8.865–16.468) (**Figure 1A**). The recurrence sites of all relapsed patients (78.9%) were explicitly documented, including 61 cases (45.2%) of intrathoracic recurrence and 74 cases (54.8%) of extra-thoracic recurrence (**Figure 2**). The median DFS in the patients with intrathoracic recurrence was 9.467 months (95%CI 6.077–12.856), slightly longer than that of patients with extra-thoracic recurrence (median DFS 9.367 months, 95%CI 7.150–11.584), but it was not statistically significant (P = 0.383) (**Figure 1B**).

According to the classification of hematogenous or lymphatic metastasis, hematogenous metastasis developed in 91 patients (67.4%), whereas lymphatic metastasis developed in 35 patients (25.9%). The remaining 9 patients (6.7%) had both hematogenous and lymphatic metastasis. The median DFS of hematogenous metastasis and lymphatic metastasis group were 9.700 months (95%CI 7.151–12.249) and 8.233 months (95%CI 6.495–9.972), respectively, which was not statistically significant (P = 0.150) (**Figure 1C**).

There were slightly more hematogenous metastasis than lymphatic metastasis (32 *versus* 29 cases) among the 61 patients with intrathoracic recurrence, with a relatively similar median DFS of 11.067 months and 8.100 months, respectively (P = 0.092) (**Figure 1D**).

Hematogenous metastasis was more than lymphatic metastasis in the 74 patients with extra-thoracic recurrence (59 *versus* 6 cases, the other 9 cases had hematogenous and lymphatic metastasis synchronously). The difference in median DFS between the two groups was not statistically significant (9.367 *versus* 8.367 months, P = 0.287) (**Figure 1E**).

Among the 68 patients with extra-thoracic recurrence in the form of hematogenous metastasis with or without lymphatic tract, there were 20 patients with brain metastasis, 19 patients with bone metastasis, and 11 patients with liver metastasis. There was 1 case of simultaneous bone and liver metastasis and 1 case of systemic metastasis. The median DFS of patients with brain, bone, and liver metastasis were 11.300 months (95%CI 2.389–20.211), 6.467 months (95%CI 5.614–7.320), and 9.367 months (95%CI 4.907–13.826), respectively (**Figure 1F**).

**Figure 3:**
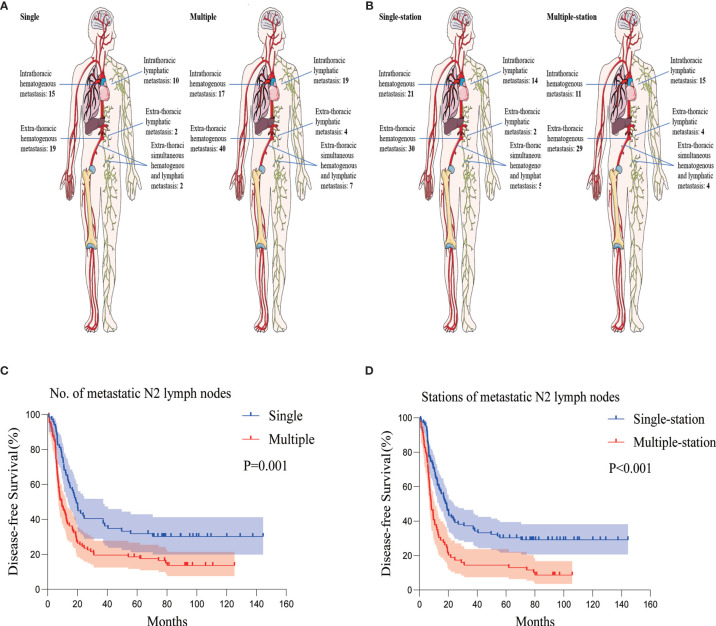
Recurrence patterns and Kaplan-Meier curves of DFS according to the number and stations of metastatic N2 lymph nodes. **(A)** Recurrence patterns of patients with single or multiple p-N2. **(B)** Recurrence patterns of patients with single- or multiple-station p-N2. **(C)** Kaplan-Meier curves of DFS for patients with single or multiple p-N2. **(D)** Kaplan-Meier curves of DFS for patients with single- or multiple-station p-N2. DFS, disease-free survival; p-N2, pathological N2.

There was no clear association between the recurrence patterns of intra- or extra-thoracic and hematogenous or lymphatic tracts among the patients with multiple p-N2, nor to the number of p-N2 stations. (**Figures 3A, B**). Patients with multiple p-N2 presented much earlier with relapse compared to those with single p-N2; the difference was not statistically significant (median DFS 9.367 *versus* 19.233 months, P = 0.001) (**Figure 3C**). The median DFS of patients with multiple-station p-N2 was significantly shorter than that of patients with single-station p-N2 (median DFS 8.100 *versus* 18.067 months, P < 0.001) (**Figure 3D**, **Supplementary Figure S1**).

According to the univariate analysis, the median DFS of patients who had received postoperative chemotherapy was significantly longer than that of those who had not [15.033 months (95%CI 10.674–19.393) *versus* 6.467 months (95%CI 2.713–10.221), P < 0.001]. The median DFS of patients who had undergone TRT was significantly longer than that of those who had not [14.533 months (95%CI 10.499–18.567) *versus* 6.400 months (95%CI 3.179–9.621), P < 0.001].

According to the multivariate analysis, we found T stage, the number of the metastatic N2 lymph nodes, postoperative chemotherapy, TRT, and PCI were independent factors for DFS after the adjustment (**Table 2**).

**Table 2:** Multivariate cox regression analysis of DFS.

Characteristic	P-value	HR (95%CI)
Gender	0.110	0.613 (0.336–1.117)
Age (years)	0.851	0.966 (0.675–1.382)
Smoking index	0.173	0.703 (0.424–1.167)
Pathologic T1	0.007	
T2	0.021	1.691 (1.083–2.640)
T3	0.134	1.524 (0.879–2.644)
T4	0.001	3.044 (1.593–5.815)
No. of metastatic N2 lymph nodes	0.000	2.500 (1.660–3.223)
Postoperative adjuvant chemotherapy	0.004	1.999 (1.240–3.223)
Thoracic radiation therapy (TRT)	0.003	2.038 (1.282–3.241)
Prophylactic cranial irradiation (PCI)	0.032	1.586 (1.042–2.415)

### OS

**Figure 4:**
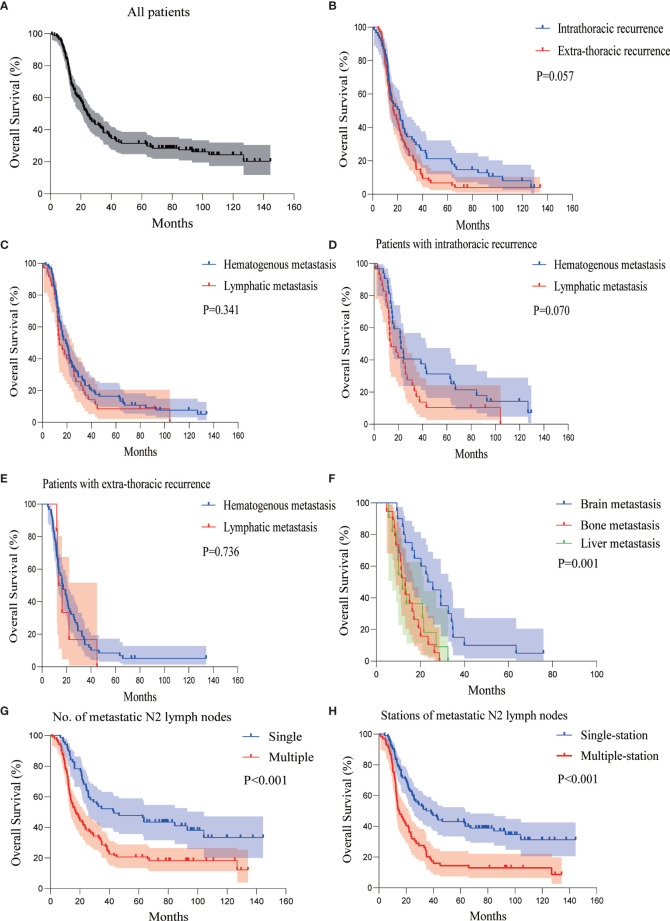
Kaplan-Meier curves of OS analysis for **(A)** all patients, **(B)** patients with intra- or extra-thoracic recurrence, **(C)** patients with hematogenous or lymphatic metastasis, **(D)** patients with intrathoracic recurrence in the form of hematogenous or lymphatic metastasis, **(E)** patients with extra-thoracic recurrence in the form of hematogenous or lymphatic metastasis, **(F)** patients with brain, bone, or liver metastasis, **(G)** patients with single or multiple metastatic N2 lymph nodes, and **(H)** patients with single- or multiple-station metastatic N2 lymph nodes. OS, overall survival; p-N2, pathological N2.

At the last follow-up, the median OS of all the patients was 24.167 months (95%CI 19.310–29.023) (**Figure 4A**). There was no statistical significance in OS between patients with intrathoracic recurrence and extra-thoracic recurrence (median OS 21.500 *versus* 16.333 months, P = 0.057) (**Figure 4B**).

Similar to DFS, the OS of hematogenous and lymphatic metastasis in all patients was not statistically significant (median OS 19.133 *versus* 14.033 months, P = 0.341) (**Figure 4C**). There was no statistically significant difference in the OS of the hematogenous and lymphatic metastasis in patients with intrathoracic recurrence (median OS 22.067 *versus* 14.033 months, P = 0.070), similar to those with extra-thoracic recurrence (median OS 17.100 *versus* 13.533 months, P = 0.736) (**Figures 4D, E**).

The median OS of patients with brain, bone, and liver metastasis was 23.400 months (95%CI 16.607–30.193), 13.100 months (95%CI 10.446–15.754), and 11.900 months (95%CI 7.800–16.000), respectively. The presence of brain metastasis was not associated with the OS (P = 0.148), while the presence of bone metastasis was associated with a significantly worse OS (P = 0.001). However, the presence of liver metastasis was associated with worse OS (P = 0.039). The brain metastasis group *versus* the bone metastasis group and brain metastasis group *versus* liver metastasis group had P < 0.001 and P = 0.006, respectively. The difference in the bone metastasis group *versus* the liver metastasis group was not significant (P = 0.554) (**Figure 4F**). This indicates that bone and liver metastasis was associated with a relatively poor prognosis compared with brain metastasis.

Analysis of the lymph node status revealed that the number and sites of metastatic p-N2 were strongly associated with OS. Similar to DFS, the OS was significantly shorter in patients with two or more p-N2 (median OS 17.100 months) compared to those with a single metastatic p-N2 (median OS 43.033 months), P < 0.001 (**Figure 4G**). The median OS of patients with single- and multiple-station p-N2 were 34.667 and 14.767 months, P < 0.001 (**Figure 4H**, **Supplementary Figure S2**).

According to the univariate analysis, the median OS of patients who had received postoperative chemotherapy and those who had not were 26.300 months (95%CI 19.043–33.557) and 14.033 months (95%CI 10.356–17.711), respectively (P = 0.004). The median OS of patients who had undergone TRT and those who had not were 25.933 months (95%CI 18.227–33.639) and 10.000 months (95%CI 3.648–16.352), respectively (P = 0.004).

According to the multivariate analysis, T stage, the number of the metastatic N2 lymph nodes, postoperative chemotherapy, TRT, and PCI were independent factors for OS after the adjustment (**Table 3**).

**Table 3:** Multivariate cox regression analysis of OS.

Characteristic	P-value	HR (95%CI)
Gender	0.328	0.732 (0.392–1.367)
Age (years)	0.545	0.891 (0.614–1.294)
Smoking index	0.558	0.856 (0.510–1.439)
Pathologic T1	0.000	
T2	0.004	2.024 (1.260–3.251)
T3	0.023	1.986 (1.101–3.585)
T4	0.000	4.050 (2.071–7.920)
No. of metastatic N2 lymph nodes	0.000	2.664 (1.750–4.053)
Postoperative adjuvant chemotherapy	0.032	1.718 (1.048–2.818)
Thoracic radiation therapy (TRT)	0.008	1.921 (1.185–3.114)
Prophylactic cranial irradiation (PCI)	0.047	1.549 (1.005–2.388)

## Discussion

The objective of the retrospective study was to observe the survival and recurrent features of p-N2 LD-SCLC patients after surgical resection. As far as we know, there are few studies in this area.

Concurrent chemoradiotherapy has been served as the cornerstone of treatment for LD-SCLC instead of surgery for decades except the selected stage I (T1-2,N0M0) patients, which is mainly based on the findings of two randomized controlled trials conducted more than 20 years ago (5, 6). As shown in CONVERT trial (13), for LD-SCLC patients who accepted concurrent chemoradiotherapy, median OS ranged from 25 to 30 months, which is not satisfying. Is there still a role for surgery in LD-SCLC patients with lymph node metastasis?

There have been few randomized clinical trials on LD-SCLC surgery in the last decade. Meanwhile, some evidence comes from prospective non-randomized studies and retrospective analysis (14–17), but they were generally conducted on few patients. Advancements in the stratification of mediastinal lymph nodes and surgical techniques call into question the applicability of surgery to modern practice.

The main recurrence site of p-N2 SCLC after surgical resection was the extra-thoracic cavity. Stish et al. (8) in 2015 investigated the recurrence patterns and long-term outcomes of LD-SCLC after surgery in 54 patients (including only 8 cases with N2); the main recurrence site was in the thoracic cavity. On the contrary, we analyzed data from 171 p-N2 LD-SCLC patients and concluded that more p-N2 patients had recurrence outside than inside the chest. One possible reason is that the sample sizes of the two studies were different. Also, we mainly observed the prognosis of p-N2 patients, while they focused on p-N0 patients (59%) and only 8 cases were p-N2. Additionally, 82.5% of patients in our study received adjuvant TRT compared to 7% of in their study, which may reduce the recurrence rate in the thoracic cavity.

Adjuvant radiotherapy is the primary locoregional treatment modality and is often followed by chemotherapy. According to a meta-analysis, adjuvant TRT could increase the local control rate of unresected LD-SCLC by 25.3% and increase the 2-year survival rate to 20% (18). There are clear benefits of radiation therapy for LD-SCLC patients with p-N2 who had undergone radical surgery (19). In our study, the 2-year survival rate and 5-year survival rate of p-N2 patients who received TRT after complete resection were approximately 54% and 34%, respectively, which were better than those who did not receive TRT (P = 0.004) with or without chemotherapy. In recent years, “the abscopal effect” in non-small cell lung cancer caused concern, referring to an underlying immune response dependent on the microenvironment playing a significant role in trigging systemic antitumor effects after receiving radiotherapy combined with immune checkpoint inhibitors (20–22). However, the abscopal effect of radiotherapy on SCLC is still in research. Whether the resected LD-SCLC patients could benefit from immune checkpoint inhibitors combined with radiotherapy or not remains unknown.

Compared with lymphatic metastasis, patients who suffered from hematogenous metastasis accounted for the majority (67.4%). Both intrathoracic and extra-thoracic recurrences were mainly hematogenous metastasis (32 out of 61 cases and 59 out of 74 cases). Local radiotherapy may reduce the chance of local lymphatic recurrence, but the results showed that hematogenous metastasis was more prone to occur, especially the distant hematogenous metastasis. Furthermore, there was no statistical difference in the OS of patients who developed hematogenous or lymphatic metastasis regardless of TRT or not (P = 0.341). The recurrence patterns do not seem to be related to the survival benefits of local radiotherapy given that p-N2 SCLC patients can benefit from postoperative local radiotherapy.

Patients suffering from bone and liver metastasis had poor prognosis and survival compared with patients suffering from brain metastasis (P = 0.001). Patients with bone metastasis had the poorest DFS (median DFS 6.467 months) with a median OS of 13.100 months. However, it was better than those suffering from liver metastasis. One reason may be that patients with bone metastasis can be treated with phosphate-containing drugs or even with local radiotherapy, which may help to achieve a relatively longer OS regardless of early recurrence. Patients with liver metastasis were associated with the poorest survival (median OS 11.900 months). There are generally few local treatments for liver metastasis, and the liver has a dual blood supply, which makes the OS worsen. The poor prognosis and survival presented by bone and liver metastasis of N2 LD-SCLC after surgical resection raise attention on monitoring and intervening in the metastasis in time.

Zhao et al. (9) analyzed the prognostic factors of LD-SCLC patients after surgical resection using multivariate analysis and found that complete resection, cigarette index, lymph node metastatic rate (the number of lymph nodes involved divided by the total number of resected lymph nodes) etc. were independent prognostic factors. However, their study regarded all postoperative patients (N0-N2). Subgroup analyses in several studies (11, 12, 23, 24) suggested that chemoradiotherapy after surgery may confer a survival advantage for patients with N2 in LD-SCLC. However, the studies did not analyze single node or single-station p-N2 and were involved small sample sizes.

Given that none of the above studies analyzed the specific p-N2 status in terms of postoperative pathology, we examined the relationship between the number, stations of p-N2, and the prognosis of the LD-SCLC patients who underwent radical resections. The results showed that the number of stations of metastatic p-N2 neither affected the first recurrence site nor did it affect hematogenous or lymphatic metastasis. However, the number of metastatic p-N2 was strongly associated with the time to recurrence (P = 0.001) and overall survival (P < 0.001). The prognosis of multiple p-N2 was poor (median DFS 9.367 months; median OS 17.100 months). Notably, the median OS of patients with single p-N2 was 43.033 months, which was significantly higher compared with concurrent chemoradiotherapy as shown in clinical trials conducted by Sundstrom et al. and Takada et al. (25, 26); however, the above studies regarded patients with LD-SCLC as a whole without specifically mentioning p-N2. This suggests a need to consider different treatment options for single and multiple p-N2 patients and shows that chemoradiotherapy as standard treatment for LD-SCLC patients with single p-N2 is debatable.

Similarly, the number of metastatic stations of p-N2 did not correlate to the recurrence patterns but was related to the time to relapse and the overall survival. Patients with multiple-station p-N2 had a worse prognosis compared with patients with single-station p-N2 (median DFS 18.067 *versus* 8.100 months, P < 0.001; median OS 34.667 *versus* 14.767 months, P < 0.001). This indicates that a favorable prognosis may occur when mediastinal lymph node metastasis is limited to a single station p-N2.

This study intended to evaluate which characteristics of patients would be crucial to the recurrence patterns of these p-N2 SCLC patients after surgery, but unfortunately, no results were obtained.

The results suggest a benefit of surgical resection for selected p-N2 LD-SCLC, especially that a favorable prognosis may occur when mediastinal lymph node metastasis is limited regardless of T stage. However, the main limitation of this study is the single-center retrospective nature, which leads to inevitable bias in data collection and patient selection. Healthier patients with fewer comorbid conditions might be disproportionally selected over sicker patients to undergo surgical resection. The potential bias and the lack of prospective validation must be considered when interpreting the results of the study.

In conclusion, in patients with p-N2 LD-SCLC who had undergone radical surgery and adjuvant therapy, the first recurrence site is extra-thoracic and hematogenous metastasis is the main form. The prognosis of bone and liver metastasis is poor. Moreover, although lymph node status seems not related to the recurrence patterns, patients with multiple p-N2 and multiple-station p-N2 may have suboptimal DFS and OS. Interestingly, LD-SCLC patients with single p-N2 exhibit good prognosis after surgical resection. Surgery may be a treatment option if LD-SCLC with a single metastatic N2 lymph node can be identified preoperatively.

## Data Availability Statement

The raw data supporting the conclusions of this article will be made available by the authors, without undue reservation.

## Ethics Statement

The studies involving human participants were reviewed and approved by the Ethics Committee of Shanghai Chest Hospital. Written informed consent for participation was not required for this study in accordance with the national legislation and the institutional requirements.

## Author Contributions

LY: data curation, formal analysis, investigation, methodology, software, roles/writing—original draft, and writing—review and editing. JX: data curation, formal analysis, investigation, methodology, and software. RQ: data curation, investigation, and methodology. HZ: project administration, supervision, validation, and visualization. BH: project administration, resources, supervision, validation, and visualization. RZ: conceptualization, data curation, project administration, resources, supervision, validation, visualization, and writing—review and editing. All authors contributed to the article and approved the submitted version.

## Funding

This work was supported by the Science and Technology Innovation Program of Shanghai [20Y11913800] and Nurture projects for basic research of Shanghai Chest Hospital [2020YNJCM04]. The funder did not participate in the study design, data collection and analysis, decision to publish, or preparation of the manuscript.

## Conflict of Interest

The authors declare that the research was conducted in the absence of any commercial or financial relationships that could be construed as a potential conflict of interest.

## Publisher’s Note

All claims expressed in this article are solely those of the authors and do not necessarily represent those of their affiliated organizations, or those of the publisher, the editors and the reviewers. Any product that may be evaluated in this article, or claim that may be made by its manufacturer, is not guaranteed or endorsed by the publisher.

## Supplementary Material

Supplementary Figure S1 Scatter plot of DFS for relapsed patients. DFS, disease-free survival. p-N2, pathological N2.

Supplementary Figure S2 Scatter plot of OS for dead patients. OS, overall survival. p-N2, pathological N2.

## Abbreviations

CI, confidence interval; DFS, disease-free survival; LD-SCLC, limited-disease small-cell lung cancer; OS, overall survival; PCI, prophylactic cranial irradiation; p-N2, pathological N2; SCLC, small-cell lung cancer; TRT, thoracic radiation therapy.
