# Supplementary figures and images for: Patterns of Recurrence and Survival Rate After Complete Resection of Pathological Stage N2 Small-Cell Lung Cancer
Source: Front Oncol. 2021 Aug 27;11:675354. doi: 10.3389/fonc.2021.675354 (PMC8429904; doi:10.3389/fonc.2021.675354)

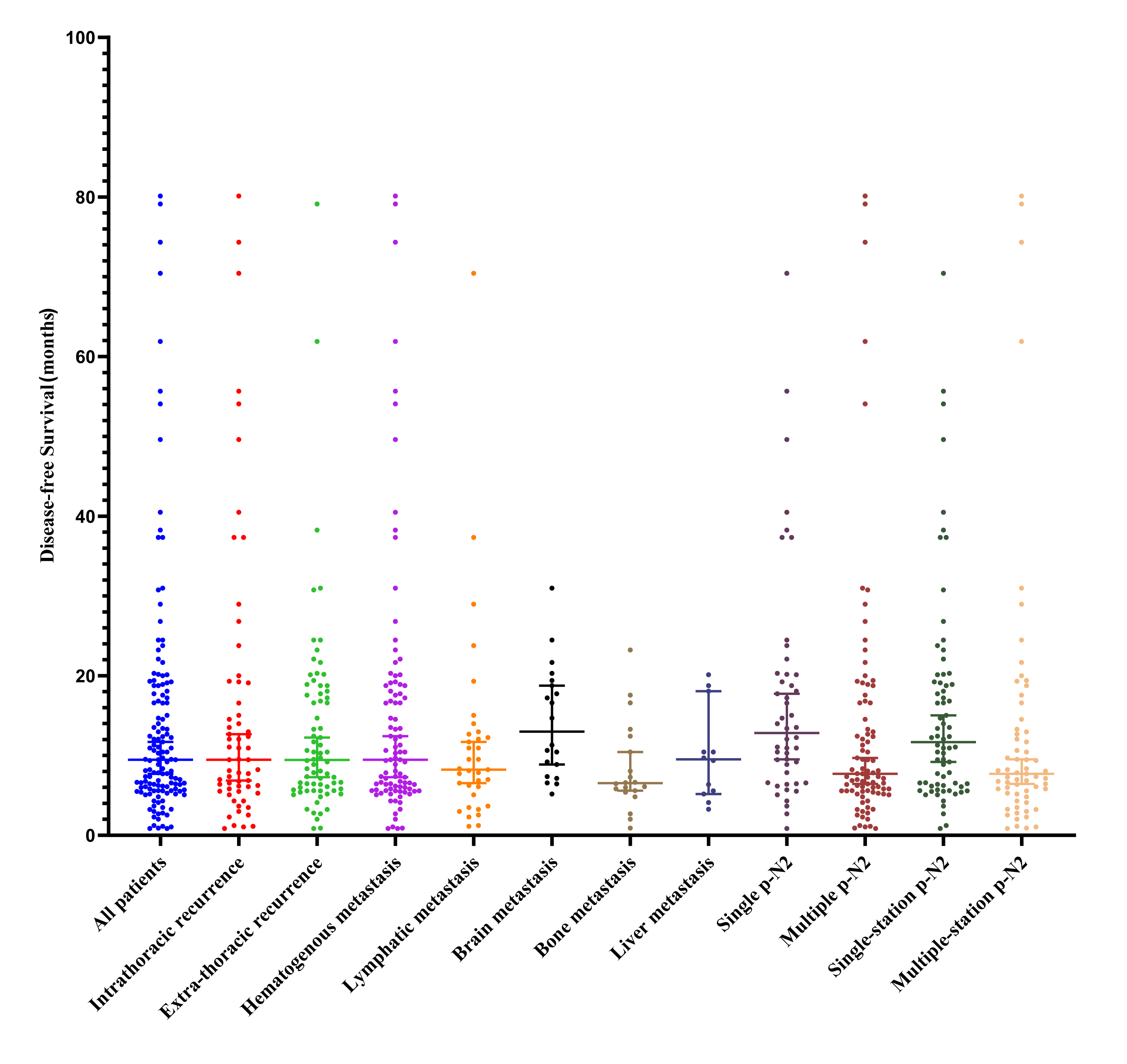

Supplement: Supplementary Figure S1 — Scatter plot of DFS for relapsed patients. DFS, disease-free survival. p-N2, pathological N2. [file Image_1.tif]

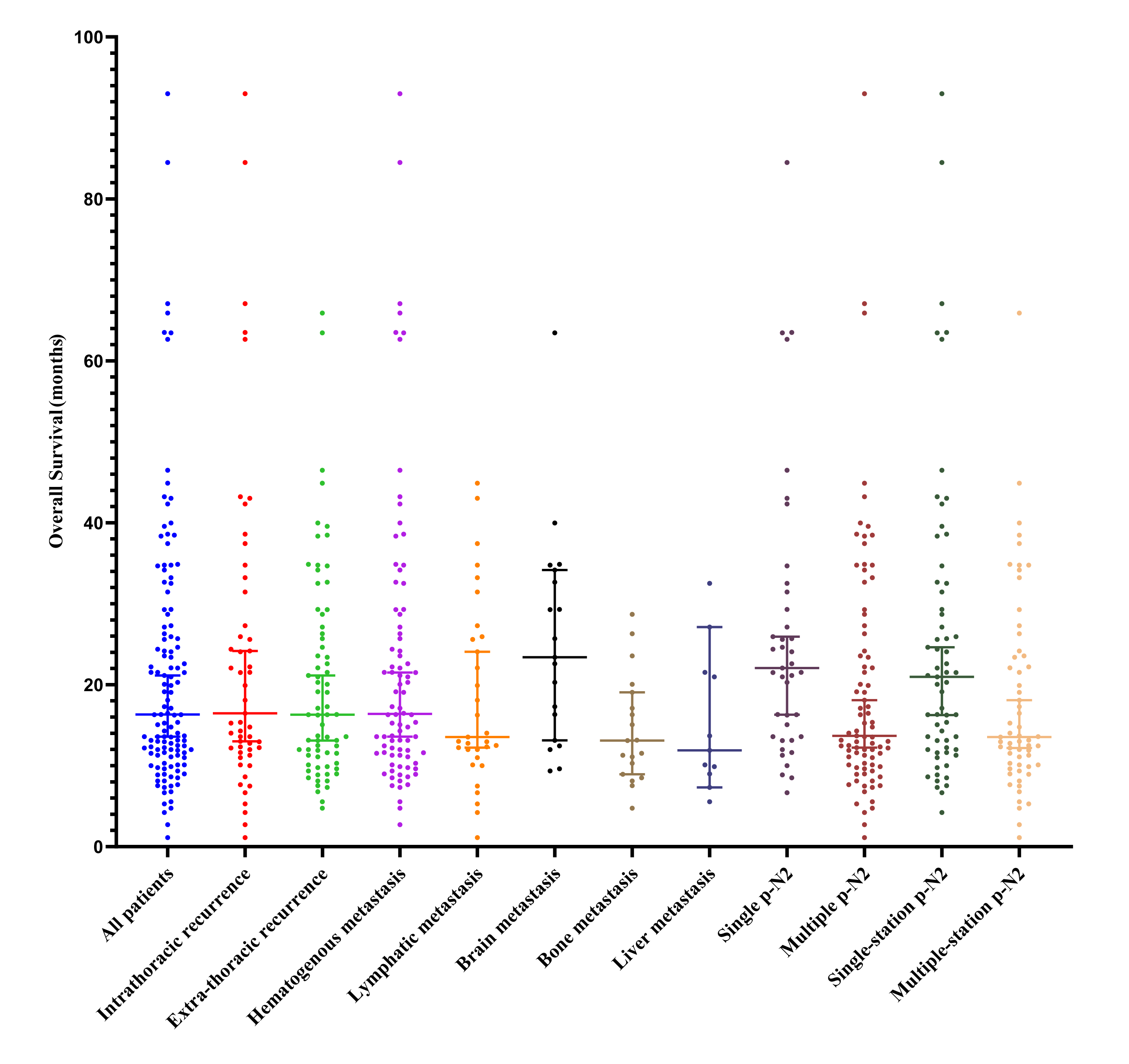

Supplement: Supplementary Figure S2 — Scatter plot of OS for dead patients. OS, overall survival. p-N2, pathological N2. [file Image_2.tif]
